# Influence of intervention treatment by “heat-clearing and diuresis-promoting” prescription on NALP3, an inflammatory factor in acute gouty arthritis

**DOI:** 10.1186/s13018-022-03046-z

**Published:** 2022-03-15

**Authors:** Jun Yu, Lianrui Li, Jie Liu, Zhiyong Chen

**Affiliations:** Department of Pharmacy, Tianjin Beichen District Chinese Medicine Hospital, No. 436 Jingjin Road, Beichen District, Tianjin, 300400 China

**Keywords:** “Heat-clearing and diuresis-promoting” prescription, Acute gouty arthritis, Inflammatory factor, NALP3, Serum uric acid

## Abstract

**Background:**

To investigate the efficacy of Qingre Lishi Decoction(QLRD), in the treatment of acute gouty arthritis, and its influence on the expression levels of inflammatory factor nucleotide-binding oligomerization domain-like receptor(NALP 3) in patients.

**Methods:**

A total of 78 patients with acute gouty arthritis admitted to our hospital were randomly divided into the control group and the observation group, with 39 cases in each group. The control group was given basic treatment and colchicine tablets, and the observation group was given “heat-clearing and diuresis-promoting” prescription for intervention treatment. The main symptom score, treatment effective rate and laboratory indexes of the two groups were compared 7 days after treatment.

**Results:**

After treatment, the scores of joint redness, hot pain, joint flexion and extension disorder, oliguria and constipation were improved in both groups, and the improvement degree in observation group was higher than that in control group (P < 0.05); the clinical effective rate in the observation group (94.87%) was higher than that in the control group (76.92%). The serum uric acid (UA), erythrocyte sedimentation rate (ESR), interleukin-1β (IL-1β) and NALP3 showed a decreasing trend, and the decrease degree of each index in observation group was higher than that in control group (P < 0.05).

**Conclusion:**

The “heat-clearing and diuresis-promoting” prescription for intervention treatment can effectively improve the clinical symptoms of patients with acute gouty arthritis and reduce the level of inflammatory factor NALP3, maintaining remarkable effect.

## Introduction

Gout is a disease which is prevalent all over the world and occurs in all age groups. The fundamental cause of this disease lies in the high level of blood uric acid in the body, which leads to deposition of uric acid crystals and results in arthritis [1]. The patients suffering from acute gouty arthritis are typically characterized by joint redness, swelling, heat and pain. With the progression of disease, the pain gradually worsens, eventually leading to diseased joint deformity and severe limitation of joint activity [2]. The disease is clinically treated by olchicine which can reduce the formation of lactic acid by inhibiting the action of white blood cells, thereby reducing the inflammatory response and controlling the progression of disease [3]. It should be noted that colchicine is prone to gastrointestinal adverse reactions, including water and electrolyte disturbance in serious cases. Some patients with chronic drug use are often intolerant, which affects the treatment effect. As an important inflammatory factor mediating gout, NALP3 can mediate the release of IL-1 and induce the occurrence of gout inflammation, which is significantly correlated with the severity of illness [4]. It is believed in traditional Chinese medicine that the main pathological mechanism of patients with acute gouty arthritis is spleen deficiency and damp heat, so the traditional Chinese medicine (TCM) with the effects of clearing heat, invigorating spleen and removing dampness may be better. The “heat-clearing and diuresis-promoting” prescription aims at removing blood stasis, clearing collaterals, dispelling pains and colds, and its good effect has been suggested in many studies [5]. However, at present, there are few clinical studies on the treatment of gout by “heat-clearing and diuresis-promoting” prescription, and it is not clear whether it can improve the treatment effect and whether it is safe and feasible. Based on this, we conducted a study on Qingre Lishi Decoction and conducted the experiment with the gout patients actually admitted to our hospital.

## Materials and methods

### General data

A total of 78 patients with acute gouty arthritis admitted to our hospital from January 2020 to December 2020 were divided into the control group and the observation group by random number method, with 39 cases in each group. There were 33 men and 6 women in the control group; aged from 32 to 70 years and with the mean age of (46.43 ± 10.98) years; the course of gout: 2 months to 8 years, with an average of (3.87 ± 1.03) years; the course of acute gout arthritis ranged from 2 to 10 days, with an average of (3.97 ± 1.42) days; BMI index: 21.8–26.1 kg/m^2^, with an average of (23.79 ± 1.68) kg/m^2^; the pathogenic site: ankle joint in 6 cases, knee joint in 2 cases, first metatarsophalangeal joint in 29 cases, and metacarpophalangeal joint in 2 cases. There were 32 men and 7 women in the observation group; aged from 31 to 70 years and with the mean age of (46.52 ± 11.01) years; the course of gout: 2 months to 9 years, with an average of (3.85 ± 1.01) years; the course of acute gout arthritis ranged from 3 to 10 days, with an average of (4.28 ± 1.12) days; BMI index: 21.5–26.8 kg/m^2^, with an average of (23.64 ± 1.47) kg/m^2^; the pathogenic site: ankle joint in 5 cases, knee joint in 1 case, first metatarsophalangeal joint in 32 cases, and metacarpophalangeal joint in 1 case. The comparison of the general data of both groups shows the differences were comparable (*P* > 0.05). This study was in compliance with relevant medical ethical standards and was approved by the Tianjin Beichen District Chinese Medicine Hospital, and all patients voluntarily participated in the study.

### Inclusion and exclusion criteria

Inclusion criteria: (1) all the patients met the diagnostic criteria under the Multidisciplinary Expert Consensus on the Diagnosis and Treatment of Hyperuricemia Related Diseases in China [6] (2017 edition); (2) no patient took non-steroidal anti-inflammatory drugs or hormones 1 week before enrollment. Exclusion criteria: (1) patients with severe organ dysfunction such as heart, brain, lung, liver and kidney; (2) patients with joint stiffness, deformity and loss of mobility; (3) patients with damaged skin or infectious diseases; (4) the patients intolerant to colchicine tablets in the control group and those intolerant to “heat-clearing and diuresis-promoting” prescription in the observation group; (5) patients with incomplete clinical data.

## Methods

The control group received oral treatment with colchicine tablets (Xishuangbanna Banna Pharmaceutical Co., Ltd., national medicine approval number H53021369), 1 mg/per time, and the dosage was one a day. Based on the control group, the observation group took “heat-clearing and diuresis-promoting” therapy incorporating edible 15 g tulip, 15 g Lysimachia christinae Hance, 10 g rhizoma atractylodis, 10 g radix clematidis, 15 g rhizoma smilacis glabrae, 10 g achyranthes root, 30 g semen coicis, 10 g golden cypress, 10 g herba lycopi, 10 g rhizoma anemarrhenae, 15 g yam rhizome and 15 g Polygonum cuspidatum. Based on the clinical symptoms of patients, 10 g golden cypress and 10 g rhizoma anemarrhenae were added to the patients with severe joint swelling, heat and pain. 10 g pangolin is for those with poor joint flexion and extension; 10 g safflower was added for blood stasis. Decoct the medicine in water, 1 dose/d, and take it in the morning and evening. Both groups were treated for 7 days as a course of treatment, and the treatment effect was evaluated when the two courses of treatment expired.

### Observation index

(1) Symptom score. The symptom improvement effect in the two groups patients was evaluated by TCM symptom score, which was formulated based on the Guiding Principles for Clinical Research of New Traditional Chinese Medicine [7], including joint redness, swelling, heat and pain, joint flexion and extension disorders, scanty dark urine and constipation. The symptoms were scored for: none—zero point, mild—2 points, moderate—4 points and severe—6 points.

(2) Treatment effective rate. The clinical efficacy in the observation group and the control group was observed. The treatment effect of the patients was divided into “excellent”: joint pain, redness, swelling, limited movement and other symptoms disappeared, and the laboratory indicators became normal; “valid”: joint pain, swelling, swelling, limited movement and other symptoms were improved, and the laboratory indicators significantly improved; “invalid”: the patient showed no significant change in clinical symptoms or symptom aggravation within 2 courses of treatment. The patients’ clinical efficacy = (“excellent” cases + “valid” cases)/total cases × 100%.

(3) Laboratory indexes. The serum uric acid (UA), erythrocyte sedimentation rate (ESR), interleukin-1β (IL-1β) and NALP3 were tested in strict accordance with the instructions of the ELISA kit, which was purchased from a biological engineering company in Shanghai.

### Statistical analysis

The statistical software SPSS21.0 was used for analysis. SPSS 22.0 (version) statistical analysis software was used. The measurement data in accordance with the normal distribution were expressed as (${\overline{\text{x}}}$ s). The t test was used for comparison between two groups, and the count data were expressed as (${\overline{\text{x}}}$ 2) rate. P < 0.05 indicated that the difference had statistical significance.

## Results

### Comparison of clinical symptom score between two groups of patients

The difference in the symptom scores of joint redness, swelling, heat and pain, joint flexion and extension disorders, scanty dark urine and constipation before treatment in both groups was not statistically significant (P > 0.05); after treatment, the symptom scores of joint redness, swelling, heat and pain, joint flexion and extension disorders, scanty dark urine and constipation showed a declining trend, and the improvement degree in observation group was higher than that in control group (P < 0.05), and the difference was statistically significant (*P* < 0.05), as shown in Table 1.

**Table 1:** Changes of scores for traditional Chinese medicine symptoms in both groups before/after treatment (points, ${\overline{\text{x}}}$ ± s)

Group	Joint redness, swelling, heat and pain		Joint flexion and extension disorders		Scanty dark urine		Constipation	
	Before treatment	After treatment	Before treatment	After treatment	Before treatment	After treatment	Before treatment	After treatment
Observation group (n = 39)	4.18 ± 0.92	1.19 ± 0.26^a^	2.75 ± 0.60	0.76 ± 0.17^a^	2.66 ± 0.59	0.68 ± 0.17^a^	2.42 ± 0.48	0.57 ± 0.17^a^
Control group (n = 39)	4.05 ± 0.88	1.84 ± 0.30^a^	2.77 ± 0.53	1.34 ± 0.28^a^	2.72 ± 0.56	1.04 ± 0.20^a^	2.46 ± 0.44	1.15 ± 0.33^a^
*t*	0.638	10.225	0.156	11.058	0.461	8.565	0.384	9.757
P	0.526	0.000	0.876	0.000	0.646	0.000	0.702	0.000

Compared with before treatment, ^a^*P* < 0.05

### Comparison of clinical efficacy between two groups of patients

**Fig. 1:**
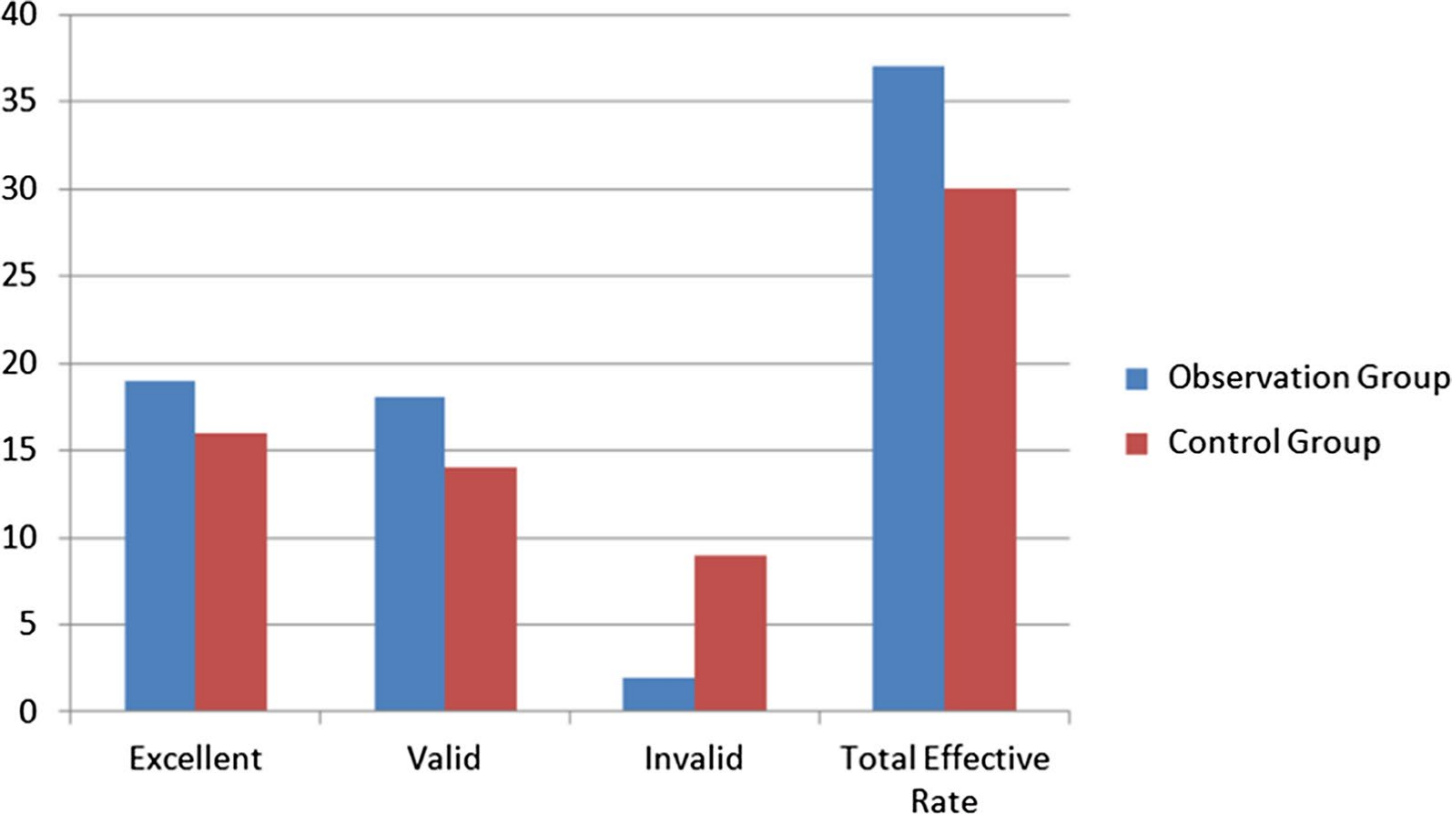
Comparison of clinical efficacy between two groups of patients

The total clinical effective rates of patients in the observation group and the control group were 94.87% (37/39) and 76.92% (30/39), respectively. The total effective rate in the observation group was higher than that in the control group, and the differences were statistically significant (× 2 = 13.297, P < 0.05). See Table 2 and Fig. 1.

**Table 2:** Comparison of clinical efficacy between two groups of patients [n, (%)]

Group	n	Excellent	Valid	Invalid	Total effective rate
Observation group	39	19 (48.72)	18 (46.14)	2 (5.13)	37 (94.87)
Control group	39	16 (41.03)	14 (35.90)	9 (23.08)	30 (76.92)
*x*^2^					13.297
*P*					0.000

Compared with before treatment, ^a^*P* < 0.05

### Comparison of laboratory indexes between two groups of patients

**Fig. 2:**
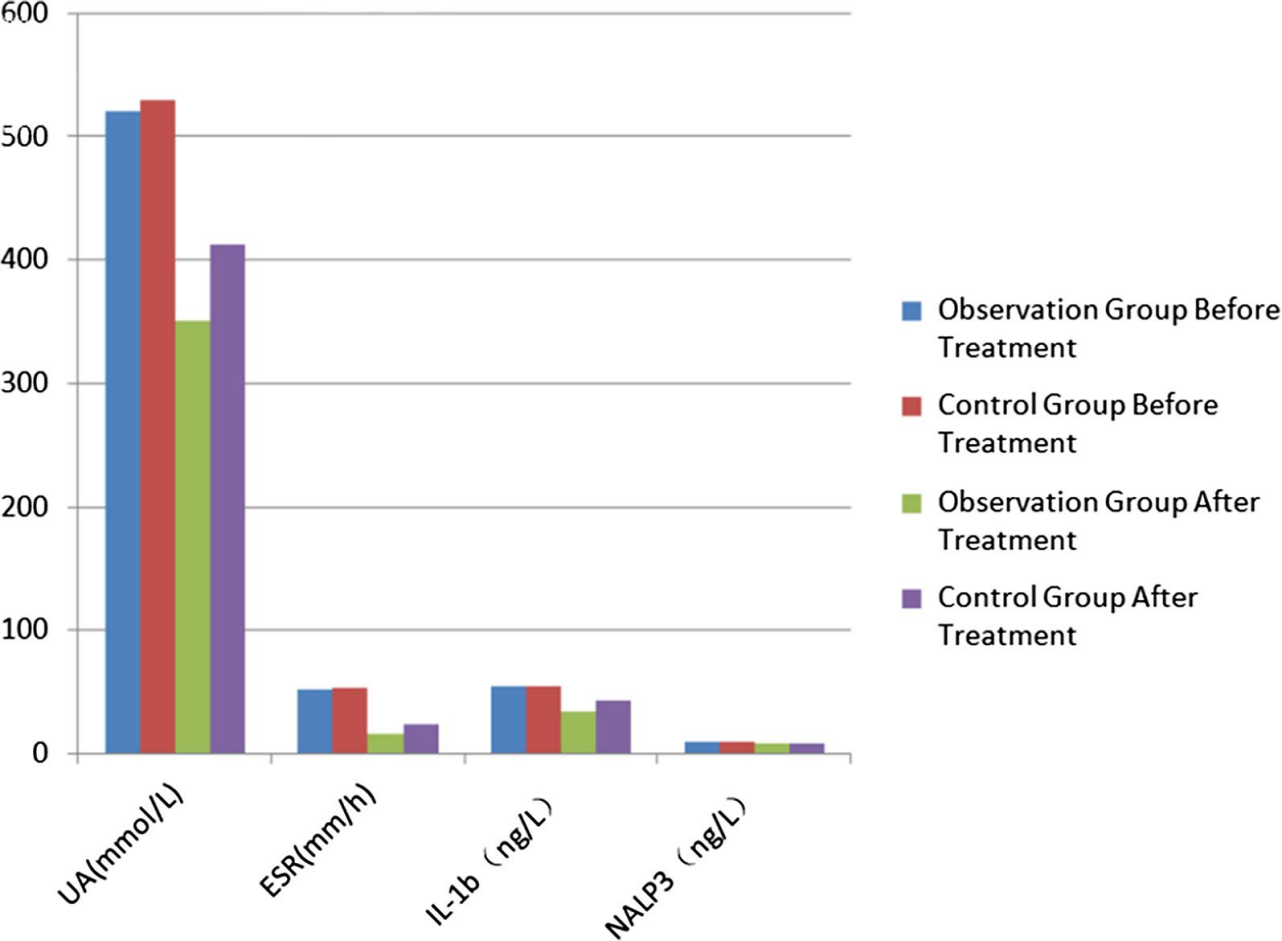
Changes of laboratory indexes in both groups before/after treatment

The difference in UA, ESR, IL-1β and NALP3 before treatment in both groups was not statistically significant (P > 0.05); after treatment, UA, ESR, IL-1β and NALP3 showed a decreasing trend, the decrease degree of each index in observation group was higher than that in control group, and the difference was statistically significant (*P* < 0.05), as shown in Table 3, Fig. 2.

**Table 3:** Changes of laboratory indexes in both groups before/after treatment (points, ${\overline{\text{x}}}$ ± s)

Group	UA (μmol/L)		ESR (mm/h)		IL-1β (ng/L)		NALP3 (ng/L)	
	Before treatment	After treatment	Before treatment	After treatment	Before treatment	After treatment	Before treatment	After treatment
Observation group (n = 39)	505.06 ± 54.97	339.20 ± 38.22^a^	45.88 ± 8.54	16.63 ± 4.85^a^	52.33 ± 9.25	32.94 ± 7.69^a^	9.46 ± 1.79^a^	7.08 ± 1.47^a^
Control group (n = 39)	515.18 ± 50.13	386.48 ± 40.69^a^	45.92 ± 8.61	22.78 ± 5.10^a^	53.50 ± 8.12	41.57 ± 8.83^a^	9.86 ± 2.14^a^	8.17 ± 1.77^a^
*t*	0.850	5.289	0.021	5.457	0.594	4.603	0.895	2.959
P	0.398	0.000	0.984	0.000	0.555	0.000	0.373	0.004

Compared with before treatment, ^a^*P* < 0.05

## Discussion

The acute gouty arthritis is common in patients with gout which will not only bring great pain to patients, but also seriously affect life and work. The positive symptomatic treatment can effectively alleviate the pain for patients and delay the progression of disease. Modern medicine mainly focuses on anti-inflammatory and analgesic treatment of patients. Although the treatment effect is good and works quickly, it is difficult to control the disease fundamentally. Meanwhile, the adverse reactions caused by some western medicines may affect the medication compliance of patients. The traditional Chinese medicine has unique advantages in the treatment of chronic diseases such as gout. Chinese medicines for clearing away heat and toxic materials, promoting blood circulation and removing blood stasis are used to clear dampness and turbidity, especially in patients with acute attack. The patients in the remission of gout need not only clear heat, remove dampness, clear turbidity and remove blood stasis, but also tonify spleen and kidney [8, 9]. Therefore, this study included the “heat-clearing and diuresis-promoting” prescription to keep the patient’s kidney essence full, healthy temper and supplementary essence, to help vital energy and blood circulation, dampness and turbidity, focusing on combination of attack and tonic, spleen and kidney treatment, and the simultaneous treatment of principal and subordinate symptoms.

With unceasing advancing theory of traditional Chinese medicine, most medical researchers and patients are now inclined to use TCM methods to treat gout. Due to the improper diet of patients, the dampness and heat are filled in, as well as the poor movement of vital energy and blood circulation, and the interaction of phlegm and heat, resulting in swelling and burning pain of the joints [10]. Qingre Lishi Decoction is an effective empirical prescription in clinic. Shā rotto Katakuri in the prescription has the effects of clearing heat and detoxicating, dissipating mass and relieving swelling. Among them, multiple alkaloids such as colchicine have good analgesic effects [11]. Rhizoma smilacis glabrae can promote uric acid excretion, remove dampness and promote joint function. Rhizoma Dioscoreae Septemlobae can dispel wind and remove impediment, effectively improve the clearance rate of endogenous creatinine and reduce blood uric acid. Clematis chinensis is mild and pungent in nature, which can significantly reduce blood uric acid, has a strong anti-inflammatory effect and effectively protects the kidney [12]. At the same time, Rhizoma Polygoni Cuspidati and Herba lycopi have the effects of clearing heat and detoxicating, expelling wind and removing dampness, as well as promoting blood circulation to remove blood stasis, removing dampness and dredging collaterals. Rhizoma anemarrhenae can purge stagnation fire, rhizoma atractylodis can dispel wind and remove dampness, and cortex phellodendri can clear heat and remove dampness. Coicis Semen, Alismatis Rhizoma and Achyranthis Radix are effective in tonifying liver and kidney, removing dampness and turbidity, activating blood and dredging channels, and regulating viscera. The results of this study showed that after the treatment, the symptom scores of joint swelling and hot pain, unfavorable joint flexion and extension, short red urine, constipation and other symptoms were all improved in the two groups. The improvement degree of patients in the observation group was higher than that in the control group. The clinical effective rate in the observation group was higher than that in the control group. This may be due to the analgesic effect of Rhizoma Atractylodis, anti-inflammatory and antipyretic effect of Rhizoma Cyperi, antipyretic effect of Radix Achyranthis bidentatae, and analgesic effect of Coicis Semen in the prescription of heat-clearing and dampness-removing formula. The combination of these drugs not only significantly inhibited joint inflammation, but also promoted uric acid metabolism [13].

The pathogenesis of acute gout joint inflammation is complex. Recent studies have found that inflammatory factors, immunoglobulins and so on play obvious roles in the pathogenesis of gout. Erythrocyte sedimentation rate refers to the natural sedimentation rate of red blood cells in the blood, which is closely related to red blood cells and plasma factors. It will be significantly increased when the body has immunological diseases, inflammatory diseases and gout. Serum uric acid is the product of purine reoxidation in the liver, and the abnormally high index is the key cause of gout. IL-1, a common subtype of interleukin -1(IL-1), can induce the production of inflammatory factors such as IL-6 and IL-8, and stimulate the synthesis of inflammatory regulatory factors to promote joint inflammatory diseases. NALP3 can effectively induce IL-1 synthesis and secretion and promote IL-1 release under the stimulation of high blood uric acid and urate crystals in the body, aggravating the inflammatory response in patients, which is closely related to the progression of gout arthritis. Studies have shown that both IL-1 and NALP3 are key factors in the inflammatory response, and their levels are significantly related to the degree of gout arthritis [14]. In this study, the average levels of UA, ESR, -1 and NALP3 water were decreased after treatment, and the level in the observation group was lower than that in the control group, indicating that Qingre Lishi Decoction could effectively reduce the acute gout arthritis caused by hyperuricemia and further reduce the content of serum uric acid. This may be because urate crystals can induce the body to produce reactive oxygen species, which can activate the LRR end of NALP3 molecule, leading to the activation of NALP3 inflammasome signaling pathway and aggravating the body's inflammatory response [15]. In the heat-clearing and dampness-removing formula group adopted in the study, Coicis Semen can reduce the level of serum uric acid by reducing the conversion rate of uric acid precursor. The giant knotweed rhizome can control the gout symptoms and regulate the erythrocyte sedimentation rate through the TLRs/My D88 pathway. The combination of these drugs can effectively inhibit the formation of urate crystals and reduce the inflammatory activator, thereby reducing the inflammatory response [16]. Due to limited conditions and small number of included samples in this study, although it was of some value, further study was required to confirm it.

## Conclusion

In summary, the heat-clearing and dampness-removing prescription given to patients with acute gout arthritis can effectively reduce pain, improve clinical symptoms, correct the level of laboratory indicators and improve clinical efficacy, which can be used for reference and implementation of treatment methods for patients with gout. This practice can be used for reference and implementation in the clinical application of such gout patients' treatment.

## Data Availability

Not applicable.

## Abbreviations

QLRD: Qingre Lishi Decoction
TCM: Traditional Chinese medicine
UA: Uric acid
ESR: Erythrocyte sedimentation rate
IL-1β: Interleukin-1β
